# Trichotillomania and Risk of Alcohol- and Drug-Related Problems

**DOI:** 10.1016/j.bpsgos.2025.100605

**Published:** 2025-09-02

**Authors:** Luis C. Farhat, Kayoko Isomura, Ralf Kuja-Halkola, Isabell Brikell, Zheng Chang, Brian M. D’Onofrio, Henrik Larsson, Paul Lichtenstein, Lorena Fernández de la Cruz, Anna Sidorchuk, David Mataix-Cols

**Affiliations:** a Department of Psychiatry, Faculdade de Medicina, Universidade de São Paulo, São Paulo, Brazil; b Centre for Psychiatry Research, Department of Clinical Neuroscience, Karolinska Institutet, Sweden, and Stockholm Health Care Services, Region Stockholm, Sweden; c Department of Medical Epidemiology and Biostatics, Karolinska Institutet, Stockholm, Sweden; d Department of Global Public Health and Primary Care, University of Bergen, Bergen, Norway; e Department of Biomedicine, Aarhus University, Aarhus, Denmark; f Department of Psychological and Brain Sciences, Indiana University, Bloomington, Indiana; g School of Medical Sciences, Örebro Universitet, Örebro, Sweden; h Department of Clinical Sciences, Lunds Universitet, Lund, Sweden

**Keywords:** Cohort studies, Disruptive, impulse control, and conduct disorders, Hair-pulling disorder, Obsessive-compulsive disorder, Registries, Substance-related disorders

## Abstract

**Background:**

Trichotillomania is an understudied psychiatric disorder characterized by repeated hair-pulling resulting in hair loss. Little is known about the risk of problematic substance use in this patient group. In this nationwide matched cohort study, we investigated the association between trichotillomania and substance-related problems.

**Methods:**

We linked various nationwide administrative and clinical registers in Sweden. Among 12,015,664 individuals living in the country from January 1, 1997, to December 31, 2020, we identified 1136 individuals with an ICD-10 diagnosis of trichotillomania at age ≥10 years (86.4% female, median age at first diagnosis 23.8 years [interquartile range 13.3–34.9 years]) and matched them with 11,360 unaffected individuals. The outcome was broadly defined as substance-related problems (alcohol- and drug-related disorders, suspected criminal offenses, and deaths). Stratified Cox proportional hazards regression was used to determine hazard ratios (HRs) for the association between trichotillomania and any substance-related problems.

**Results:**

Over a mean follow-up period of 6 years, substance-related problems were recorded for 137 (12.1%) individuals with trichotillomania and 399 (3.5%) matched individuals (crude incidence rates per 1000 person-years of 21.6 and 5.6, respectively). After controlling for sociodemographic covariates and parental substance-related problems, trichotillomania was associated with an increased relative risk of substance-related problems (HR, 3.12; 95% CI, 2.53–3.85). Adjusting also for comorbid psychiatric history did not meaningfully change the findings.

**Conclusions:**

Individuals with trichotillomania had a 3-fold increased risk of substance-related problems compared with unaffected individuals. Future research should examine the mechanisms underlying this association and inform the clinical management of the dual diagnoses.

## Plain Language Summary

Trichotillomania is a psychiatric disorder characterized by recurrent hair-pulling behavior that results in noticeable hair loss, despite repeated efforts to reduce or stop the behavior. In this study, we examined whether individuals diagnosed with trichotillomania in Swedish specialist care had an elevated risk of substance-related problems, including alcohol- and drug-related disorders, suspected criminal offenses, and mortality. Using a cohort of 1136 individuals with trichotillomania and 11,360 matched unaffected control individuals, the analyses revealed a 3-fold increased relative risk of substance-related problems in individuals with trichotillomania over a mean follow-up period of 6 years. These findings underscore the importance of screening for substance use issues when assessing individuals with trichotillomania.

Trichotillomania (also known as hair-pulling disorder) is a psychiatric disorder defined by repetitive pulling out of one’s own hair, despite attempts to stop or cut down on the behavior, that results in noticeable hair loss and embarrassment, shame, feelings of loss of control, and/or impairment in socio-occupational functioning (1).

Both major classification systems, the DSM and the ICD, currently place trichotillomania in the same chapter as obsessive-compulsive disorder. Impulsivity and compulsivity were initially described as opposite ends of a spectrum (2), but these dimensional traits are positively correlated (3), and neurocircuitry implicated in one may overlap or interact with that implicated in the other (4). Consistently, the core symptoms of hair-pulling disorder also share phenomenological similarities with psychiatric disorders characterized by impulsivity (5). For example, about two-thirds of individuals with trichotillomania may experience tension before and relief after hair pulling (6), which is common in impulse control disorders (7). Additionally, previous studies have shown that trichotillomania may be associated with impaired motor inhibition (8), impulsive decision making (9), and high rates of co-occurring impulse control disorders (10,11). At the same time, at least some hair-pulling behaviors may be done to regulate internal stimuli (12), and hair-pulling symptoms may correlate with emotional regulation difficulties in individuals with trichotillomania (13).

Gratification and compensation play an important role in the development and maintenance of addictive behaviors (14), and some have posited that trichotillomania may be conceptualized as a behavioral addiction, akin to gambling disorder (15). However, previous research examining whether individuals with hair-pulling disorder are at increased risk of substance-related problems is scarce and has yielded disparate findings. A review of 6 U.S. studies examining comorbidity rates of trichotillomania reported that, on average, 17% of individuals with hair-pulling disorder may experience a substance use disorder (SUD) during their lifetime (16). More recently, one study examined current psychiatric comorbidities self-reported by 175 individuals with trichotillomania ascertained from the general population through convenience sampling via the internet and found that 18% of them indicated an alcohol use or a drug use disorder (10). In another study from the same group, it was found that 13% of 121 individuals with hair-pulling disorder recruited from the community experienced past-year harmful drinking as suggested by Alcohol Use Disorders Identification Test (AUDIT) scores (17). Altogether, those rates are not distinctively different from corresponding estimates in the U.S. general population for the lifetime prevalence of SUDs (15%), past-year SUDs (17%), or alcohol use disorders (10%) (18,19).

Comparisons of rates of substance-related problems in individuals with trichotillomania and individuals from the general population across studies are hampered by differences in substance use rates and behaviors across populations, as well as methodological variations between studies. Additionally, previous studies examining rates of SUDs or of harmful alcohol use and drug use among individuals with trichotillomania ascertained participants through nonprobabilistic survey designs or research-active clinics, relied on self-reported information without thorough diagnostic assessments, and/or adopted a retrospective assessment of lifetime substance use behaviors in one-time surveys (20). These shortcomings limit the extent to which definitive conclusions can be drawn from currently existing data. Large cohort studies with prospective follow-ups, clinician diagnoses, and a broader definition of substance-related problems including events that are independent of help-seeking behaviors (e.g., substance-related deaths or suspected criminal offenses) could provide much-needed evidence on the issue of whether individuals with trichotillomania are at increased risk of substance-related problems.

To address this clear research gap, in this study, we leveraged the Swedish nationwide registers to test the hypothesis that individuals diagnosed with trichotillomania in specialist services would have an increased risk of alcohol- and drug-related morbidity, criminality, and mortality.

## Methods and Materials

This study was approved by the Swedish Ethical Review Authority (Reference No. 2020-06540). Informed consent was not required because the study was based on the nationwide registers, and the included individuals were not identifiable at any time.

### Data Sources

The personal identity number, a unique numeric code that is assigned to all individuals who are born in Sweden or immigrate to the country (21), was used to link multiple nationwide registers, including 1) the Census Register, which contains sociodemographic information on all Swedish residents since 1960; 2) the Total Population Register, which holds demographic and migration data since 1968; 3) the Longitudinal Integration Database for Health Insurance and Labour Market Studies (LISA), with annually updated demographic and socioeconomic data since 1990; 4) the National Patient Register (NPR), which covers diagnostic information on inpatient (since 1969 and 1973 for somatic and psychiatric conditions, respectively) and outpatient specialized (since 2001) care visits; 5) the Cause of Death Register, which contains information on deaths since 1952; 6) the Prescribed Drug Register (PDR), which contains information on prescribed medications dispensed in all pharmacies in Sweden since July 2005; 7) the Register of People Suspected of Offences, which contains records of individuals age ≥15 years who were suspected of offenses after the conclusion of an investigation since 1995; 8) the Multi-Generation Register, with information on kinship for people born in Sweden (since 1932) or registered in the country (since 1961); and 9) the Halmstad University Register on Pupils with Intellectual Disability (HURPID), which contains records on pupils who graduated from an upper secondary school for students with an intellectual disability between 2000 and 2020 (22, 23, 24, 25, 26, 27, 28).

### Study Population

We identified all individuals who lived in Sweden at any time from January 1, 1997 (the date of the implementation of the ICD-10 in Sweden), to December 31, 2020 (the end of the study period). From this population, we excluded individuals who died or emigrated before their 10th birthday or who were born after December 31, 2010 (i.e., were younger than 10 years of age by the end of the study) because we considered that records of substance-related problems would be unlikely prior to this age. Furthermore, to minimize the risk of diagnostic misclassification, we excluded individuals with a diagnosis of intellectual disability in the NPR (ICD-10 F70–F79) or who completed upper secondary school for pupils with intellectual disabilities according to the HURPID (29,30).

### Exposure

Individuals were considered exposed if they had at least 1 inpatient or specialist outpatient diagnosis of trichotillomania (ICD-10 code F63.3) registered in the NPR (as either primary or secondary diagnosis) at age ≥10 years between 1997 and 2020. The sociodemographic and clinical characteristics of individuals with a diagnosis of trichotillomania in speciality services in Sweden are similar to those reported in other countries (29).

Exposed individuals entered the cohort on the date of their first diagnosis of trichotillomania (i.e., the index date). Each exposed individual was matched on sex, birth year, and county of residence on the index date with 10 randomly selected individuals from the study population who did not have a diagnosis of trichotillomania by the index date.

### Outcome

The outcome was any substance-related problem, comprising 1) inpatient or specialist outpatient diagnoses of alcohol or drug use disorders, including accidental poisoning (as either primary or secondary diagnosis), identified using ICD-10 codes from the NPR; 2) alcohol- or drug-related suspected criminal offenses, identified through the corresponding codes in the Register of Persons Suspected of Offences; and 3) deaths due to alcohol or drug use disorders, including accidental poisoning (as either underlying or secondary causes), identified with ICD-10 codes in the Cause of Death Register. To maximize the identification of alcohol and drug use disorders, we also included dispensed medications used in the treatment of alcohol dependence and opioid use disorders, respectively, recorded with the corresponding Anatomical Therapeutic Chemical Classification codes in the PDR. All included codes are listed in Table S1.

We adopted a broad definition of substance-related problems instead of solely relying on diagnoses of alcohol or drug use disorders identified from the NPR because in Sweden less severe cases of alcohol- or drug-related disorders are not referred to specialist services (31), and diagnoses made in primary care settings are not recorded in the NPR (24). Furthermore, there is an underutilization of treatment services for alcohol or drug use disorders. To partially overcome this limitation, and consistent with previous literature (32,33), we also considered deaths and suspected criminal offenses related to alcohol or drug use in our outcome definition, because those events are independent of help-seeking behaviors and utilization of the health care system.

### Covariates

In addition to variables used for matching, we collected information on place of birth (Sweden or abroad) from the Total Population Register and several sociodemographic variables measured on the index date or the nearest year available from the Census Register and LISA. This included data on the highest attained educational level (elementary [≤9 years], secondary [10–12 years], or higher education [>12 years]), civil status (single/divorced/widowed or married/cohabiting), and disposable family income level (lowest 20%, middle 60%, or top 20%).

The Multi-Generation Register was used to identify biological parents. For maternal and paternal lifetime substance-related problems, as well as for the personal history of substance-related problems prior to the index date, we considered the same definition and registers that were used for the outcomes and extracted additional data from earlier ICD editions (ICD-8 and ICD-9 codes), if available (Table S1).

For history of psychiatric disorders, we used the NPR to extract information on the following groups of diagnoses, recorded before or on the index date: neurodevelopmental disorders, psychotic disorders, bipolar disorders, depressive disorders, anxiety- and stress-related disorders, obsessive-compulsive disorder, eating disorders, and emotionally unstable personality disorder (see ICD codes in Table S2).

### Follow-Up

All individuals were followed from the index date until the date of the outcome, emigration, death by non-outcome, or the end of the study period (i.e., December 31, 2020), whichever occurred first. If matched unaffected individuals received a diagnosis of trichotillomania during the follow-up, they were censored on the date of their first trichotillomania diagnosis.

### Statistical Analyses

The main analyses comprised all individuals, regardless of the presence of preexisting substance-related problems, to ensure generalizability of the findings to the real world because about 10% to 15% of the general population have lifetime substance use morbidity (18,34). All analyses were performed for any substances pooled together and then separately for alcohol and other drugs.

We fit stratified Cox proportional hazards regression, with the matched clusters treated as separate strata, to estimate hazard ratios (HRs) and 95% CIs for the association between trichotillomania and substance-related problems. Missing data were coded as unknown for sociodemographic covariates and parental history of substance-related problems and included in the models as nominal variables.

A minimally adjusted model (model 1) only controlled for matching variables (i.e., sex, birth year, and county of residence). Subsequent models also adjusted for other sociodemographic characteristics (i.e., place of birth, highest attained educational level, civil status, and disposable family income level) (model 2) and additionally for lifetime parental (maternal and paternal) substance-related problems (model 3).

We repeated the 3 models stratified by the type of the first outcome event, i.e., whether the first outcome record was a clinical event (comprising a diagnosis of alcohol or drug use disorders, including poisoning, or a dispensed medication used in the treatment of alcohol dependence or opioid use disorders), a suspected criminal offense, or death.

Additionally, because trichotillomania is more common in females than in males (35), the 3 models were repeated separately by sex. In addition, to explore the influence of history of psychiatric disorders on the associations, we repeated model 2 with additional adjustment for different groups of psychiatric disorders, one at a time. We chose model 2 for these additional analyses to avoid missing data on parental substance-related problems.

Furthermore, we repeated the 3 models in the subcohort restricted to individuals without preexisting substance-related problems to ensure that the measured outcomes would represent incident cases. Lastly, to avoid potential misclassification with medications used for pain relief, we planned to repeat the main analyses in the whole cohort after excluding dispensation records of medications used for opioid use disorders from the outcome definition (codes in Table S1).

## Results

### Matched Cohort Description

**Figure 1:**
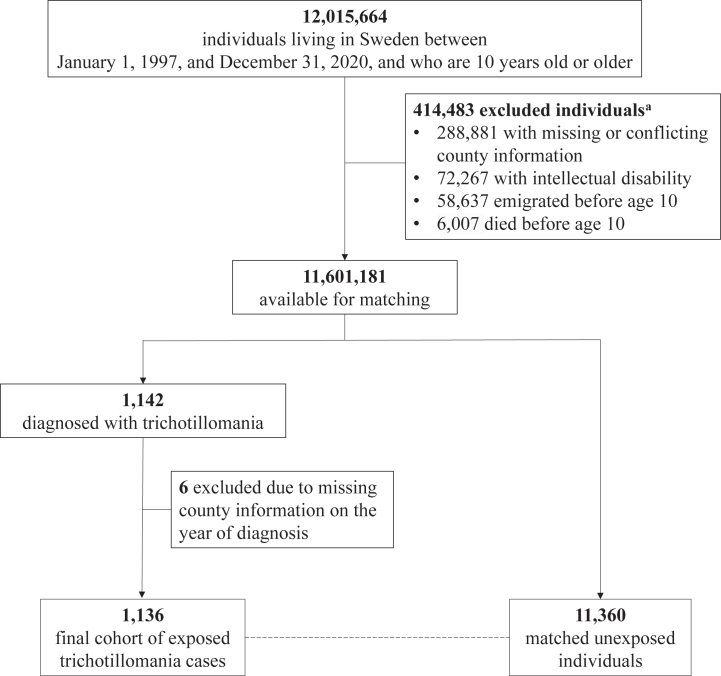
Selection of individuals with a diagnosis of trichotillomania and matched unaffected individuals. ^*a*^The numbers of excluded individuals in each category add up to a number larger than the total because some individuals were excluded for more than one reason.

Of 12,015,664 individuals who were living in Sweden from January 1, 1997 to December 31, 2020, 11,601,181 were eligible for inclusion in the cohort (Figure 1). Among them, 1136 individuals had a diagnosis of trichotillomania, with a median age at first recorded diagnosis of 23.8 years (interquartile range 13.3–34.9) and a mean follow-up time of 5.6 years (SD 4.6). Those individuals were matched to 11,360 unaffected individuals, who were followed up for a mean of 6.3 years (SD 5.0).

The characteristics of study participants on the index date are summarized in Table 1. Individuals with a diagnosis of trichotillomania were more likely to be less educated, single, and have a lower disposable family income than unaffected individuals. They were also more likely to have a mother with substance-related problems, a personal history of alcohol- and drug-related problems, and a history of psychiatric disorders compared with the unaffected individuals.

**Table 1:** Characteristics of Study Participants at the Time of Matching

	Individuals With Trichotillomania, *n* = 1136	Matched Unaffected Individuals, *n* = 11,360	χ^2^	*p*
Year of Birth				
≤1939	29 (2.5%)	290 (2.5%)	NA	NA
1940–1959	63 (5.6%)	630 (5.6%)		
1960–1979	250 (22.0%)	2500 (22.0%)		
1980–1999	590 (51.9%)	5900 (51.9%)		
≥2000	204 (18.0%)	2040 (18.0%)		
Age on the Index Date^a^, Years	23.8 [13.3–34.9]	23.8 [13.3–34.9]		
Sex				
Female	982 (86.4%)	9820 (86.4%)	NA	NA
Male	154 (13.6%)	1540 (13.6%)		
Place of Birth				
Sweden	941 (82.8%)	9200 (81.0%)	2.31	.13
Abroad	195 (17.2%)	2160 (19.0%)		
Highest Attained Educational Level				
Elementary	414 (36.4%)	3480 (30.6%)	16.56	.001
Secondary	346 (30.5%)	3688 (32.5%)		
Higher	307 (27.0%)	3428 (30.2%)		
Unknown	69 (6.1%)	764 (6.7%)		
Civil Status				
Single, divorced, or widowed	962 (84.7%)	8466 (74.5%)	64.32	<.0001
Married or cohabiting	135 (11.9%)	2500 (22.0%)		
Unknown	39 (3.4%)	394 (3.5%)		
Disposable Family Income Level				
Lowest 20%	334 (29.4%)	1988 (17.5%)	104.93	<.0001
Middle 60%	599 (52.7%)	6561 (57.8%)		
Top 20%	163 (14.4%)	2360 (20.8%)		
Unknown	40 (3.5%)	451 (3.9%)		
Lifetime Maternal Substance-Related Problems				
Yes	74 (6.5%)	544 (4.8%)	6.92	.03
No	929 (81.8%)	9388 (82.6%)		
Unknown^b^	133 (11.7%)	1428 (12.6%)		
Lifetime Paternal Substance-Related Problems				
Yes	129 (11.4%)	1163 (10.2%)	1.43	.49
No	840 (73.9%)	8530 (75.1%)		
Unknown^b^	167 (14.7%)	1667 (14.7%)		
Preexisting Alcohol-Related Problems	90 (7.9%)	316 (2.8%)	86.82	<.0001
Preexisting Drug-Related Problems	99 (8.7%)	243 (2.1%)	167.75	<.0001
History of Any Psychiatric Comorbidities				
Neurodevelopmental disorders	284 (25.0%)	332 (2.9%)	1074.1	<.0001
Psychotic disorders	42 (3.7%)	60 (0.5%)	128.1	<.0001
Bipolar disorders	60 (5.3%)	75 (0.7%)	206.4	<.0001
Depressive disorders	441 (38.8%)	531 (4.7%)	1678.6	<.0001
Anxiety- and stress-related disorders	358 (31.5%)	492 (4.3%)	1203.7	<.0001
Obsessive-compulsive disorder	217 (19.1%)	59 (0.5%)	1651.1	<.0001
Eating disorders	100 (8.8%)	173 (1.5%)	256.12	<.0001
Emotionally unstable personality disorder	81 (7.1%)	65 (0.6%)	384.65	<.0001
Duration of Follow-Up, Years	5.6 (4.6)	6.3 (5.0)	4.6^c^	<.0001

Values are presented as *n* (%), median [interquartile range], or mean (SD).

NA, not applicable.

a The index date corresponds to the date of the first diagnosis of trichotillomania among exposed individuals and the corresponding date among matched unexposed individuals.

b Individuals for whom linkage to mothers and/or fathers was not performed due to missing data on parents in the Multi-Generation Register.

c Measured by *t* test for continuous variables.

### Risk of Substance-Related Problems

There were 137 (12.1%) individuals with trichotillomania and 399 (3.5%) matched unexposed individuals who had records of any substance-related problem during the follow-up period (crude incidence rates [IRs] per 1000 person-years of 21.6 and 5.6, respectively). Most exposed and unexposed individuals had a clinical event as their first outcome record. We could not analyze death as a separate outcome because there were fewer than 5 such first outcome events for exposed and/or unexposed individuals (omitted to ensure anonymity) (see Table S3). The 2 groups did not differ significantly on the age at which they had their first record of a substance-related problem (mean [SD] = 30.8 [11.5] vs. 29.5 [13.3], *t*_534_ = −0.99, *p* = .3).

**Figure 2:**
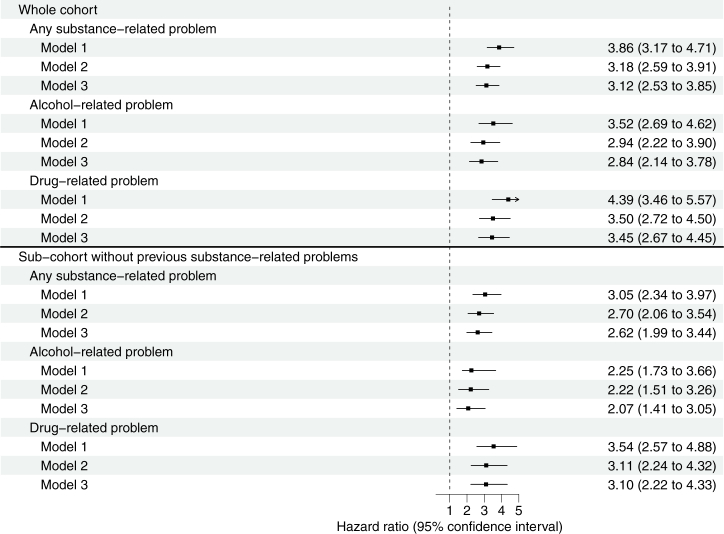
Associations between trichotillomania and substance-related problems in the entire cohort (top) and in a subcohort excluding individuals with preexisting substance-related problems (bottom). Note: Model 1 corresponds to a minimally adjusted model (i.e., sex, birth year, county of residence). Model 2 corresponds to additional adjustment (in addition to model 1) for sociodemographic characteristics (i.e., place of birth, highest attained educational level, civil status, disposable family income level). Model 3 corresponds to additional adjustment (in addition to model 2) for parental substance-related problems (maternal and paternal).

The minimally adjusted model indicated that individuals with trichotillomania had a nearly 4-fold higher relative risk of any substance-related problems (HR, 3.86; 95% CI, 3.17–4.71) compared with matched unaffected individuals. HRs were only slightly attenuated after additional adjustment for place of birth and sociodemographic characteristics (model 2: HR, 3.18; 95% CI, 2.59–3.91) and also for parental substance-related problems (model 3: HR, 3.12; 95% CI, 2.53–3.85). Similar increases in risks across all models were observed for alcohol- and drug-related problems when they were analyzed separately (Figure 2).

When considering models stratified by the type of the first outcome event, there was evidence of an increased relative risk of alcohol-related (HR, 3.14; 95% CI, 2.33–4.24) and drug-related (HR, 4.98; 95% CI, 3.55–6.98) clinical events. There was also evidence of an increased relative risk of drug-related (HR, 2.03; 95% CI, 1.32–3.12), but not alcohol-related (HR, 1.35; 95% CI, 0.48–3.78), suspected criminal offenses (Table S3).

In analyses stratified by sex, all findings remained statistically significant within males and females. Although the magnitude of the associations varied between females and males, the differences across sex should be interpreted with caution due to overlapping CIs and limited statistical power (Table 2).

**Table 2:** Associations Between Trichotillomania and Substance-Related Problems, Separately for Males and Females

	Individuals With Trichotillomania, *n* = 1136	Matched Unaffected Individuals, *n* = 11,360	Model 1	Model 2	Model 3
	*n* (%)	*n* (%)	HR (95% CI)	HR (95% CI)	HR (95% CI)
Any Substance-Related Problems					
Females	111 (11.3%)	313 (3.2%)	3.92 (3.14–4.88)	3.28 (2.61–4.13)	3.27 (2.60–4.13)
Males	26 (16.9%)	86 (5.6%)	3.65 (2.32–5.73)	3.14 (1.93–5.12)	2.96 (1.79–4.88)
Alcohol-Related Problems					
Females	53 (5.4%)	187 (1.9%)	3.04 (2.23–4.14)	2.67 (1.94–3.67)	2.62 (1.90–3.62)
Males	19 (12.3%)	35 (2.3%)	6.38 (3.58–11.4)	4.41 (2.30–8.47)	4.63 (2.32–9.27)
Drug-Related Problems					
Females	79 (8.0%)	181 (1.8%)	4.67 (3.57–6.11)	3.71 (2.79–4.92)	3.78 (2.83–5.05)
Males	19 (12.3%)	62 (4.0%)	3.50 (2.07–5.92)	3.05 (1.73–5.38)	2.89 (1.62–5.16)

Model 1 corresponds to a minimally adjusted model (i.e., sex, birth year, county of residence). Model 2 corresponds to additional adjustment (in addition to model 1) for sociodemographic characteristics (i.e., place of birth, highest attained educational level, civil status, disposable family income level). Model 3 corresponds to additional adjustment (in addition to model 2) for parental substance-related problems (maternal and paternal).

HR, hazard ratio.

Further adjustment for history of psychiatric disorders (1 group at a time) slightly attenuated the results of the main analyses; however, the increase in risk remained statistically significant for all outcomes of interest (Table 3).

**Table 3:** Associations Between Trichotillomania and Substance-Related Problems After Additional Adjustment for Comorbid Psychiatric History

	NDDs	Psychosis	Bipolar Disorders	Depressive Disorders	Anxiety- and Stress-Related Disorders	OCD	Eating Disorders	Emotionally Unstable Personality Disorder
Any Substance-Related Problem	2.58 (2.07–3.22)	3.11 (2.53–3.83)	3.05 (2.47–3.76)	2.08 (1.65–2.62)	2.52 (2.03–3.15)	3.09 (2.46–3.88)	3.03 (2.46–3.74)	2.79 (2.25–3.47)
Alcohol-Related Problem	2.53 (1.88–3.41)	2.91 (2.20–3.86)	2.81 (2.12–3.74)	2.08 (1.52–2.84)	2.33 (1.73–3.14)	2.79 (2.05–3.79)	2.80 (2.11–3.73)	2.50 (1.85–3.37)
Drug-Related Problem	2.71 (2.06–3.56)	3.38 (2.62–4.37)	3.41 (2.64–4.39)	2.18 (1.63–2.91)	2.81 (2.15–3.67)	3.54 (2.68–4.67)	3.30 (2.55–4.27)	3.22 (2.48–4.17)

Values are presented as hazard ratio (95% CI). All analyses are based on model 2 with additional adjustment for specific disorder group (1 group at a time).

NDDs, neurodevelopmental disorders; OCD, obsessive-compulsive disorder.

The subcohort restricted to individuals without preexisting substance-related problems included 983 individuals with a diagnosis of trichotillomania (86% of 1136) and 10,874 unaffected individuals (96% of 11,360). Incident events of substance-related problems were recorded among 74 (7.5%) exposed and 288 (2.6%) unexposed individuals (crude IR per 1000 person-years of 12.9 and 4.2, respectively). For all outcomes, the significantly increased risks persisted in all models and were similar but slightly lower than those reported for the whole cohort (Figure 2).

Because our data exploration indicated that no cohort members had their first outcome event defined by dispensation records of medications used for opioid use disorders, we did not perform the planned sensitivity analysis excluding those data from the outcome definition.

## Discussion

To our knowledge, this is the first nationwide population-based study to examine the association between trichotillomania diagnosed in specialist services and the risk of broadly defined substance-related problems. Over a mean follow-up period of 6 years, we observed that individuals with a diagnosis of trichotillomania had an approximately 3-fold increased risk of substance-related problems compared with matched unaffected individuals. The increased risk remained substantial and statistically significant after further adjustments for potential confounders, such as sociodemographic characteristics and parental substance-related problems, and when restricting analyses to the subcohort of individuals without a prior substance-related problem. Importantly, when we further adjusted for psychiatric history, the magnitude of observed risks was attenuated but remained statistically significant, indicating that the observed risk of substance-related problems in trichotillomania was not fully explained by psychiatric comorbidity.

Our results complement those of some previous cross-sectional studies based on population-based or help-seeking samples (10,16,17,36,37), which reported conflicting findings regarding an increased risk of SUDs or harmful alcohol use and drug use in trichotillomania. Potential reasons for the discrepant findings are manifold. Regarding the ascertainment of participants, previous studies relied on convenience samples recruited locally or through the internet, whereas our study relied on nationwide health care records. Regarding the outcome definition and its measurement, previous studies considered lifetime or current self-reported comorbidities and answers to questions about past-year alcohol use behaviors (e.g., AUDIT scores), whereas our study considered real-world diagnoses from health care services, as well as events that did not depend on treatment-seeking behaviors (e.g., suspected criminal offenses).

The identification of a strong association between trichotillomania and substance-related problems naturally leads to the question of what factors contribute to the development of harmful alcohol use or drug use in the context of hair-pulling disorder. On the one hand, this association may be indicative of an underlying difficulty inhibiting behavioral responses to urges, such as those to pull hair, drink alcohol, or use drugs. Empirical evidence has consistently indicated that individuals with trichotillomania show response inhibition deficits and an impulsive pattern in decision making (8,9). Furthermore, reward system dysfunctions have been implicated in trichotillomania. For example, Snorrason *et al.* (38) showed that cue-triggered urges and appetitive motivation to pull hair were associated with hair-pulling severity in a sample of a few hundred individuals with trichotillomania. Additionally, Grant *et al.* (39) demonstrated that individuals with trichotillomania had hyperactivation of brain regions involved in reward processing when anticipating reward or punishment in one functional magnetic resonance imaging task-based study. Another study also showed higher impulsivity among individuals with trichotillomania who reported past-year hazardous alcohol use (17). Lastly, there is some evidence that SUDs may be more common in first-degree relatives of individuals with trichotillomania (40), which could be indicative of shared genetic factors related to dysfunctions in the reward system, although no study to date has directly focused specifically on this question. On the other hand, some hair-pulling behaviors termed focused or internally regulated (12,41) are thought to be driven by negative affect (e.g., feelings of anxiety, sadness, or frustration), and some individuals may engage in hair-pulling, drinking, or drug use behaviors as coping strategies. Previous studies have consistently indicated that individuals with trichotillomania may have high levels of neuroticism and emotional regulation difficulties (42,43) and may perceive their use of alcohol or drugs as being driven by the negative feelings associated with hair pulling (44). The findings from the current study reinforce the need for future research to examine the role of both positive and negative valence systems in trichotillomania and the development of co-occurring substance-related problems.

From a clinical perspective, our findings suggest that practitioners should carefully monitor their patients with trichotillomania for alcohol or drug use. In general, organizations such as the American Academy of Child & Adolescent Psychiatry, the American Academy of Pediatrics, the UK National Institute for Health and Care Excellence, the U.S. National Institute on Alcohol Abuse and Alcoholism, the U.S. Preventive Services Task Force, and the World Health Organization recommend that practitioners ask questions about unhealthy alcohol or drug use during routine assessments of adolescents and adults (45, 46, 47, 48, 49, 50, 51). Our findings reinforce that this recommendation should be followed closely by those who see patients with trichotillomania. In particular, special attention should be devoted to adolescents because, for most individuals, trichotillomania may onset during this period (52), which represents a window of vulnerability to addictive behaviors (53). Additionally, practitioners may wish to learn whether, and how, the treatment of individuals with trichotillomania should be modified in the context of SUDs. However, there are no data to inform this clinical conundrum, as most trichotillomania treatment studies have excluded participants with co-occurring SUDs. We hope that our findings will foster more clinical research studies on the management of individuals who present with both trichotillomania and SUDs.

Our study has several strengths, including the nationwide coverage of Swedish registers and the prospective and uniform data collection procedures. To the best of our knowledge, our study is the largest and longest longitudinal study in the field, as previous prospective investigations have been conducted in the context of treatment studies with small samples to monitor maintenance of treatment response after trials of psychotherapeutic or pharmacological interventions (54, 55, 56, 57).

Our study also has limitations. First, as we have discussed elsewhere (29), only a fraction of individuals with trichotillomania are currently diagnosed in Swedish specialist services. Therefore, our findings may not generalize to individuals with trichotillomania who do not seek help for their hair pulling (e.g., milder forms of trichotillomania). At the same time, previous evidence suggests that trichotillomania symptom severity may not be significantly different among people with versus without hazardous alcohol use (17). The validity of the trichotillomania code in the Swedish registers has not been formally examined (e.g., through the evaluation of medical records). Nevertheless, we previously showed that the characteristics of individuals with a diagnosis of trichotillomania in the Swedish registers are remarkably similar to those of individuals with a diagnosis of trichotillomania from other countries (29). Thus, our findings should be applicable to patients with trichotillomania seen in specialist services in other parts of the world. Similarly, the individuals identified as having an alcohol or drug use disorder in the NPR likely represent severe cases because a considerable proportion of individuals with those diagnoses only receive treatment in primary care services in Sweden (31). Although the validity of ICD codes for substance-related morbidity and mortality (which were part of our outcome definition) has not been directly examined, evidence from other Nordic countries suggests that there is acceptable agreement between codes for SUDs and diagnoses based on structured interviews (58). Second, we cannot dismiss the possibility that individuals with a diagnosis of trichotillomania were at increased risk of receiving co-occurring diagnoses of alcohol- or drug-related disorders because they underwent neuropsychiatric assessments in the health care system (i.e., ascertainment bias). Third, we cannot guarantee that missing data on sociodemographic covariates (for 3%–7% of the study participants) and parental substance-related problems (for 15% of participants) are unrelated to the outcome, which could affect the results of analyses in which those variables were used for further adjustment (models 2 and 3). To partially overcome this limitation, we coded missing data as a separate category (unknown) and also controlled for parental substance-related problems in a separate model. Lastly, given that there are differences in the prevalence rates of substance use behaviors across countries (59,60), future studies in other contexts with different demographic compositions and baseline rates of substance-related problems would be helpful to evaluate whether the association between trichotillomania and substance-related problems is similar cross-nationally.

### Conclusions

Individuals with trichotillomania had a 3-fold increased risk of any substance-related problems compared with matched unaffected individuals. These findings underline the need for future research on the mechanisms underlying the observed association and on the clinical management of this dual psychopathology.

## Supplementary Material

Tables S1–S3

Key Resource Table
