# Supplementary material for: Trichotillomania and Risk of Alcohol- and Drug-Related Problems
Source: Biol Psychiatry Glob Open Sci. 2025 Sep 2;5(6):100605. doi: 10.1016/j.bpsgos.2025.100605 (PMC12547216; doi:10.1016/j.bpsgos.2025.100605)
Supplement: Tables S1–S3 [file mmc1.pdf]

SUPPLEMENTARY INFORMATION

Trichotillomania and Risk of Alcohol- and Drug-Related Problems

Farhat *et al.*

Supplementary Table S1. Outcome definition, data sources and extracted data.

Group	Domain	Register	Codes used for data collection
Alcohol-related problems	Alcohol use disorders, accidental poisoning, and deaths due to alcohol use	National Patient Register and Cause of Death Register	**ICD-8**: 291; 303; 980,00; 980,01<br>**ICD-9**: 291; 303, 305A; 980A<br>**ICD-10**: F10; T51; X45
	Medications used in the treatment of alcohol dependence	Prescribed Drug Register	**ATC**: N07BB01; N07BB03; N07BB04; N07BB05
	Alcohol-related suspected criminal offences	Register of People Suspected of Offences	**Criminal offences codes**: 3005[a]; 3201[b]
Drug-related problems	Drug use disorders, accidental poisoning, and deaths due to drug use	National Patient Register and Cause of Death Register	**ICD-8**: 304; 971<br>**ICD-9**: 292; 304, 305X; 969<br>**ICD-10**: F11-16; F18; F19; T40; T42; X41; X42
	Medications used in the treatment of opioid use disorders	Prescribed Drug Register	**ATC**: N07BC01; N07BC02; N07BC05; N07BC51
	Drug-related suspected criminal offences	Register of People Suspected of Offences	**Criminal offences codes**: 3070[c]; 5010[d]; 5011[e]; 5012[f]

a – Driving under the influence of only alcohol, or both alcohol and drugs (from the Law 1951:649, paragraphs 4 and 4a)
b – Operating maritime vessel under the influence of alcohol or other drugs (from the Law 1994:1009, chapter 20, paragraphs 4 and 5)
c – Driving under the influence of only drugs; from the Law 1951:649, paragraphs 4 and 4a
d – Possession of drug only; from the Narcotic Drug Act 1968:64
e – Use of drug only; from the Narcotic Drugs Act 1968:64
f – Possession and use of drugs; from the Narcotic Drugs Act 1968:64
Abbreviations: ATC = Anatomical Therapeutic Chemical Classification; AUD = Alcohol use disorders; ICD = International classification of diseases.

Supplementary Table S2. International Classification of Diseases (ICD) codes used to identify psychiatric diagnoses in the National Patient Register.

Group of psychiatric disorders (if applicable)	Psychiatric disorders	ICD codes	Minimal age to retrieve records of diagnoses
Neurodevelopmental disorders	Attention-deficit/ hyperactivity disorder[a]	**ICD-9**: 314W, 314X<br>**ICD-10**: F90	≥ 3 years
	Pervasive developmental disorders	**ICD-9**: 299A<br>**ICD-10**: F84	≥ 1 year
	Tourette syndrome or chronic tic disorders[b]	**ICD-8**: 306.2<br>**ICD-9**: 307C<br>**ICD-10**: F95	≥ 3 years
	Psychotic disorders	**ICD-8**: 295 (minus 295.5), 297, 298 (minus 298.09)<br>**ICD-9**: 295 (minus 295F), 297, 298 (minus 298A)<br>**ICD-10**: F20, F21, F22, F23, F24, F25 (minus F25.0), F28, F29	≥ 10 years
	Bipolar disorders	**ICD-8**: 296 (minus 296.00 and 296.2)<br>**ICD-9**: 296 (minus 296B)<br>**ICD-10**: F25.0, F30, F31, F34.0	≥ 10 years
	Depressive disorders (major depressive disorder, persistent mood disorder, and unspecified mood disorder)	**ICD-8**: 296.0, 296.2, 298.09<br>**ICD-9**: 296B, 298A, 300E, 311<br>**ICD-10**: F32, F33, F34 (minus F34.0), F38, F39	≥ 6 years
Anxiety- and stress-related disorders	Phobic and anxiety disorders	**ICD-8**: 300.0, 300.2,<br>**ICD-9**: 300A, 300C<br>**ICD10**: F40, F41	≥ 6 years
	Reaction to severe stress and adjustment disorders	**ICD-8**: 307, 308.4<br>**ICD-9**: 308, 309<br>**ICD-10**: F43	≥ 6 years
	Obsessive-compulsive disorder	**ICD-8**: 300.3<br>**ICD-9**: 300D<br>**ICD-10**: F42	≥ 6 years
	Eating disorders	**ICD-9**: 307B, 307F<br>**ICD-10**; F50.0-F50.3, F50.9	≥ 8 years
	Emotionally unstable personality disorder	**ICD-10**: F60.3	≥16 years

a – Individuals with attention-deficit/hyperactivity disorder (ADHD) were also identified by prescription of ADHD drugs, collected from the Prescription Drug Register, specifically amphetamine (Anatomical Therapeutic Chemical [ATC] Classification System code: N06BA01), dexamphetamine (N06BA02), methylphenidate (N06BA04), atomoxetine (N06BA09), and lisdexamfetamine (N06BA12).
b – Tourette syndrome and chronic tic disorder cases were ascertained as based on the algorithm described in Rück et al. (2015).

Supplementary Table S3. Associations between trichotillomania and substance-related problems, separately for specific types of outcome events

	Individuals with trichotillomania (n=1,136)		Matched unaffected individuals (n=11,360)		Model 1		Model 2		Model 3	
	N	%	N	%	HR	95% CI	HR	95% CI	HR	95% CI
**Alcohol-related problems**										
No outcomes	1,064	93.7	11,138	98.0						
Clinical event[a]	67	5.9	191	1.7	3.82	2.88 to 5.07	3.21	2.39 to 4.32	3.14	2.33 to 4.24
Suspected criminal offence	5	0.4	30	0.3	1.79	0.69 to 4.65	1.38	0.51 to 3.79	1.35	0.48 to 3.78
Death	NA	NA	NA	NA						
**Drug-related problems**										
No outcomes	1,038	91.4	11,117	97.9						
Clinical event[a]	68	6.0	116	1.0	6.48	4.76 to 8.80	5.00	3.59 to 6.96	4.98	3.55 to 6.98
Suspected criminal offence	29	2.5	123	1.1	2.54	1.69 to 3.82	2.13	1.39 to 3.26	2.03	1.32 to 3.12
Death	NA	NA	NA	NA						

Note: Model 1 corresponds to minimally adjusted model (i.e., sex, birth year, county of residence). Model 2 corresponds to additional adjustment (on top of Model 1) for sociodemographic characteristics (i.e., place of birth, highest attained educational level, civil status, disposable family income level). Model 3 corresponds to additional adjustment (on top of Model 2) for parental substance-related problems (maternal and paternal). NA denotes less than 5 events in the cell, and therefore the results were omitted to ensure anonymity.
a – denotes both alcohol use disorders and medications used in the treatment of alcohol dependence (or drug use disorders and medications used in the treatment of opioid use disorder)
